# Classification of Structural MRI Images in Alzheimer's Disease from the Perspective of Ill-Posed Problems

**DOI:** 10.1371/journal.pone.0044877

**Published:** 2012-10-10

**Authors:** Ramon Casanova, Fang-Chi Hsu, Mark A. Espeland, for the Alzheimer's Disease Neuroimaging Initiative

**Affiliations:** Department of Biostatistical Sciences, Wake Forest School of Medicine, North Carolina, United States of America; University of Manchester, United Kingdom

## Abstract

**Background:**

Machine learning neuroimaging researchers have often relied on regularization techniques when classifying MRI images. Although these were originally introduced to deal with “ill-posed” problems it is rare to find studies that evaluate the ill-posedness of MRI image classification problems. In addition, to avoid the effects of the “curse of dimensionality” very often dimension reduction is applied to the data.

**Methodology:**

Baseline structural MRI data from cognitively normal and Alzheimer's disease (AD) patients from the AD Neuroimaging Initiative database were used in this study. We evaluated here the ill-posedness of this classification problem across different dimensions and sample sizes and its relationship to the performance of regularized logistic regression (RLR), linear support vector machine (SVM) and linear regression classifier (LRC). In addition, these methods were compared with their principal components space counterparts.

**Principal Findings:**

In voxel space the prediction performance of all methods increased as sample sizes increased. They were not only relatively robust to the increase of dimension, but they often showed improvements in accuracy. We linked this behavior to improvements in conditioning of the linear kernels matrices. In general the RLR and SVM performed similarly. Surprisingly, the LRC was often very competitive when the linear kernel matrices were best conditioned. Finally, when comparing these methods in voxel and principal component spaces, we did not find large differences in prediction performance.

**Conclusions and Significance:**

We analyzed the problem of classifying AD MRI images from the perspective of linear ill-posed problems. We demonstrate empirically the impact of the linear kernel matrix conditioning on different classifiers' performance. This dependence is characterized across sample sizes and dimensions. In this context we also show that increased dimensionality does not necessarily degrade performance of machine learning methods. In general, this depends on the nature of the problem and the type of machine learning method.

## Introduction

In the past, it has been argued that when classifying brain structural MRI (sMRI) images, the performance of machine learning techniques is greatly affected by the curse of dimensionality (CoD). The term CoD was introduced by Richard Bellman while working on optimization problems [1]. He pointed out that some problems become intractable as the number of the variables increases. For example, a Cartesian grid in 10 dimensions with spacing of 0.1 on the unit cube will have 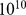 points, 20 dimensions lead to 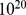 points, and so on. He concluded that if the goal was to estimate a function on a grid generated from a few dozen dimensions, one would need to evaluate it trillions of times, rendering the problem intractable. In other words, as the dimension increases, the number of samples needed to keep the same density grows exponentially. A consequence is that it is only feasible to sample high-dimensional spaces very sparsely; this is known as the empty space phenomenon [2]. CoD effects also occur in different technical areas such as function estimation, numerical integration, and machine learning. In the context of machine learning applications, it usually refers to the degradation in performance of machine learning algorithms with the increase of dimension. In such cases, researchers typically adopt approaches to first reduce the dimensionality of data before applying machine learning algorithms, as is the common practice in the field of early prediction of Alzheimer's disease (AD) using neuroimaging data. Examples include the Spatial Pattern of Abnormalities for Recognition of Early AD (SPARE-AD) and Structural Abnormality Index (STAND), based on classifiers estimated using sMRI images. In these cases, the dimensions of the original voxel space is first markedly reduced using approaches such as image processing operations [3], [4], region of interests (ROI) [5], [6], principal components analysis (PCA) [7], or ROI with *a priori* knowledge [8], [9].

Recent work provides evidence that dimension reduction for classifying brain MRI images may not be necessary to achieve a high level of prediction performance in some situations. Reports in the fMRI literature [10], [11] and especially those related to classification of structural MRI images using ADNI data, provided evidence that linear classifiers may be robust to increased dimensionality. Several researchers [12]–[14] solved classification problems of large size (up to 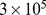) using linear kernel support vector machines (SVMs). The use of kernel methods implies a dimension reduction, since the optimization problem is solved in the space spanned by the kernel which is of dimension equal to the sample size [15]. Recently, an important contribution towards understanding the role of sample size and feature selection in the context of neuroimaging high-dimensional problems was made by Chu and colleagues [8], [14]. Using ADNI data, they studied the performance of the linear kernel SVM method [12] combined with different strategies for feature selection and sample size; most previous studies, by contrast, operated with fixed sample sizes. The study included the high-dimensional case of selecting 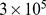 voxels by thresholding a gray matter (GM) image template. In this last situation, there was very little improvement in classification accuracy when feature selection was combined with the linear SVM. In addition, they found that feature selection based on *a priori* knowledge, such as selecting only the voxels falling in the hippocampal and parahippocampal tissue, produced significantly greater classification accuracies.

We have recently proposed solving similar structural MRI classification problems associated with AD following a different path, which is based on large-scale regularization. We use regularized logistic regression (RLR) based on a coordinate-wise descent technique as implemented in the GLMNET library [16]. Unlike other approaches these classification methods operate directly in the voxel space using regularization with sparsity properties. In previous work using ADNI data we varied the number of variables between 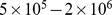 by using different study customized templates [17], [18] while keeping the sample size fixed (N = 98), and obtained excellent prediction performance and interpretability. Different versions of regularization methods with sparsity properties have been applied before in the context of fMRI data analysis to solve problems of much lower dimensions. For example, elastic net regularized least squares have been applied to problems of prediction of purchases [19] and to analyze the Pittsburgh-EBC-Group (PBAIC) competition data using Least Angle Regression optimization [20]. Ryali and colleagues extended the coordinate-descent method proposed by [21] to implement RLR with L_1_ penalty to the elastic net case [11]. They applied it to discriminate between musical and speech stimuli. In all these cases, analyses were carried out with fixed sample sizes and input spaces based on region of interest (ROI) data or a voxel space with fixed dimension on the order of 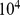.

Most of the methods described above that are capable of handling sMRI or fMRI high dimensional data are related, in one way or another to regularization theory. Regularization techniques have been widely adopted by machine learning researchers because of their capacity to deal with problems with small sample sizes and large numbers of variables, situations for which traditional statistical techniques are not well suited [22], [23]. Regularization discovery is usually credited to Tikhonov, who developed it while working on numerical solutions to integral equations [24]. He proposed it to deal with “ill-posed” problems [25]. The great French mathematician Hadamard defined at the beginning of the 20th century [26] a well-posed problem as one whose solution fulfilled three properties: 1) existence, 2) uniqueness and 3) a continuous dependence on the data (stability). He wrongly postulated the non-existence of practical problems that did not follow his definition of well-posed. However, ill-posed problems (those that break one of Hadamard's three properties) are commonly found in many scientific areas such as astronomy, tomography, image processing, oil and mine prospecting, brain research and genetics. The basic idea behind Tikhonov's regularization paradigm was to minimize a functional composed of two terms: 1) a loss term, which drives the fidelity of the solution to the patterns present in the data, and 2) a regularization or penalty term, constraining the solution to some predefined functional spaces with specific smoothness properties. In addition, a regularization parameter controls the tradeoff between these two criteria. According to Vapnik the discovery of the regularization principles by Tikhonov, Ivanov, Phillips and others was one of the more important discoveries that led to a revolution in data analysis and the creation of statistical learning theory [27]. Several important learning machines, such as RLR, SVM, and ridge regression, are considered to be particular cases of the original Tikhonovian paradigm, where the loss and the regularization terms take different forms with respect to the original Tikhonov functional [28]–[30]. For example, SVM is associated with the so-called hinge loss function, while the binomial deviance and least square loss functions are characteristic of the RLR and the ridge regression, respectively. Different types of penalties generate different versions of these learning machines.

Although regularization methods are often applied to deal with the sMRI image classification problems, it is rare to find in the literature an analysis of this problem ill-posedness, even though regularization techniques were first created to deal with the ill-posedness of practical problems. The present study provides a view of the classification of MRI images from the perspective of linear ill-posed problems. For these the degree of ill-posedness is linked to the conditioning and structure of singular values of the linear kernel matrices [31], [32]. We study the impact of conditioning of linear kernel matrices across sample sizes and dimensions on the behavior of three linear classifiers when solving neuroimaging problems of very high dimensions. Our analyses evaluate the impact of dimension (number of variables) and sample size on linear kernel matrix conditioning and their relationship to linear learning machines that operate directly in the voxel space. We provide a detailed performance evaluation of RLR with sparsity regularization implemented via coordinate descent techniques across sample sizes and dimensions showing that it is not only practical for solving these high-dimensional problems in neuroimaging but also renders excellent results in terms of prediction performance comparing very well with SVM approaches [12] previously used by other researchers. In addition, we study the linear regression classifier (LRC) [10] to help elucidate why these machine learning methods are less affected by the CoD than may be expected. Finally, we compare the performance of these techniques with counterparts based on principal components analysis (PCA), a very popular dimension reduction technique in neuroimaging applications [5], [33] and biomedical applications in general.

## Materials and Methods

### ADNI database

Data used in the preparation of this article were obtained from the Alzheimer's Disease Neuroimaging Initiative (ADNI) database (adni.loni.ucla.edu). The ADNI was launched in 2003 by the National Institute on Aging (NIA), the National Institute of Biomedical Imaging and Bioengineering (NIBIB), the Food and Drug Administration (FDA), private pharmaceutical companies and non-profit organizations, as a $60 million, 5-year public private partnership. The primary goal of ADNI has been to test whether serial magnetic resonance imaging (MRI), positron emission tomography (PET), other biological markers, and clinical and neuropsychological assessment can be combined to measure the progression of mild cognitive impairment (MCI) and early Alzheimer's disease (AD). Determination of sensitive and specific markers of very early AD progression is intended to aid researchers and clinicians to develop new treatments and monitor their effectiveness, as well as lessen the time and cost of clinical trials. The Principal Investigator of this initiative is Michael W. Weiner, MD, VA Medical Center and University of California – San Francisco. ADNI is the result of efforts of many coinvestigators from a broad range of academic institutions and private corporations, and subjects have been recruited from over 50 sites across the U.S. and Canada. The initial goal of ADNI was to recruit 800 adults, ages 55 to 90, to participate in the research. These would include approximately 200 cognitively normal older individuals to be followed for 3 years, 400 people with MCI to be followed for 3 years and 200 people with early AD to be followed for 2 years. For up-to-date information, see www.adni-info.org.

### ADNI Participants

We used ADNI subject data collected from 50 clinic sites that had their own individual IRB approval. Study subjects gave written informed consent at the time of enrollment for data collection, storage and research and completed questionnaires approved by each participating sites' Institutional Review Board (IRB). The data were anonymized before being shared. We used baseline structural MRI data from 727 subjects. Of these, 205 were cognitively normal controls (CN), 171 had AD, and 351 had mild cognitive impairment (MCI) at baseline [34]. The MCI subjects were included only to generate the study- customized template. In previous work, we observed increases in classifiers' performance when MCI subjects were also used to generate the study-customized template [18]. All classification analyses were carried out using only CN and AD participants. To reduce noise, we decided before the experiments to discard 17 subjects who were CN at baseline but converted to other cognitive status during the 36 months follow-up, leaving 188 CN subjects. Demographic information for the ADNI participants used in the analyses was briefly summarized in Table 1.

**Table 1:** Demographic data of the ADNI participants used in this study.

Cognitive Status	Number	Mean Age (std)	Sex (M/F)	Mean MMSE (std)
CN	205	76.1 (5.0)	112/93	29.1 (1.0)
MCI	351	75.1 (7.3)	228/123	27.1 (1.8)
AD	171	75.5 (7.7)	95/76	23.4 (2.0)

### Structural MRI data processing

We used baseline 1.5 T T1-weighted MRI data as described in the ADNI acquisition protocol [35]. The ADNI protocol acquires 2 sets of structural data at each visit. These are rated for image quality and artifacts by ADNI investigators [35]. To enhance standardization across sites and platforms, the best quality data set then undergoes additional pre-processing, including corrections for gradient non-linearity [36] and intensity non-uniformity [37]. In the present study, these optimally pre-processed images were downloaded from the ADNI database and used for subsequent analyses. The images were segmented and normalized using the Statistical Parametric Mapping (SPM) software package. Segmentation of the original images into gray matter (GM), white matter (WM), and cerebrospinal fluid (CSF) images was performed using the NewSegment tool. Normalization was carried out using Diffeomorphic Anatomical Registration using Exponentiated Lie algebra (DARTEL) method [38]. First, a study-customized template was generated of the 727 images using the default parameters. Second, GM, WM, and CSF images were then warped to the template, modulated and smoothed using an isotropic Gaussian kernel of 4 mm. The final resolution of the images was 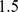 mm isotropic. Only the GM images were used in this study.

### Regularization methods in the GLMNET library

We evaluated the performance of logistic regression with elastic net regularization as implemented in the GLMNET library [16]. Our software implementation is based on MATLAB, where the GLMNET library is called using a freely available MATLAB wrapper developed by Hui Jiang (http://www-stat.stanford.edu/~tibs/glmnet-matlab/). The general form of the optimization problem solved by the library is of the form:
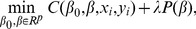
(1)

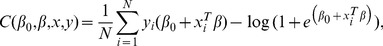
(2)

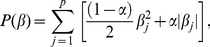
(3)where 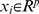 is the i^th^ sample or feature vector containing the GM voxels entering the analysis, 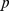 is the number of voxels entering the analysis, 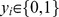 is the i^th^ label (0 for cognitively normal participants, 1 for participants with Alzheimer's disease), 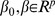 are the parameters of the model, and 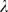 is the regularization parameter. The regularization scheme described by Eq.(1) contains two terms: a loss term 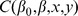 and a penalty term 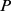 called elastic net penalty, which is a linear combination of L_1_ and L_2_ penalties. The regularization parameter 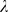 establishes a trade-off between the two terms in Eq.(1) while the regularization parameter 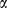 regulates the weight of the two penalties in Eq.(3). Both parameters are estimated from the data as explained below.

### Support Vector Machines (SVM)

This is the most common classification technique used to analyze sMRI data [3], [12], [38]–[41]. There are many sources describing in detail the principles behind SVM [42], [43]; we refer the reader to those while here we briefly describe our implementation and also provide an equivalent representation based on the representer theorem [44] that will be referred later to in our discussion.

Kloppel and colleagues used hard-margin (HM) and soft margin (SM) linear SVM to classify structural MRI images [12], [45]. We follow a similar approach to the SM-SVM model, using the kernel approach based on the LIBSVM library [46] implementation. GM images after normalization and thresholding are vectorized and treated as samples. A linear kernel matrix 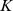 is generated by computing the inner products across all examples. This is provided to the LIBSVM library as a pre-computed kernel. For the SM-SVM the optimization of parameter C was carried out using the CV described below.

SVM based on the representer theorem and the properties of the reproducing kernel Hilbert spaces [30], [44] can be considered a regularization method. It can be represented as:
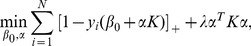
(5)where 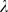 plays the role of regularization parameter, K is the linear kernel 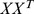, 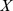 is a 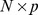 matrix containing in each row the imaging information corresponding to one subject and 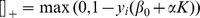 is the hinge loss function. Alternatively, using the eigen decomposition of K (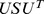) it can be written as:
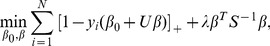
(6)where U is a square matrix containing the eigenvectors and S a diagonal matrix containing the eigenvalues. Eqs.(5–6) describe the dependence of a linear SVM on the linear kernel matrix and its singular values. Note that in this case the SVM penalizes more strongly contributions coming from the eigenvectors associated with smaller eigenvalues. The penalty in Eq.(6) has been called the generalized ridge penalty [43]. Note also that K is symmetric and therefore positive semi-definite which implies that singular and eigenvalues decompositions are the same.

### Linear regression classifier

The linear regression classifier is estimated by solving the least squares problem:
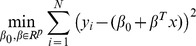
This has the general analytical solution
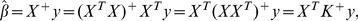
(7)where+indicates Moore-Penrose pseudo-inverse, 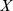 is a 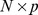 matrix containing in each row the imaging information corresponding to one subject, 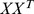 is the linear kernel matrix, 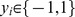 and 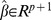. Transformations in Eq.(7) are based on well-known properties of the pseudo-inverse [47], [48]. Similar to the linear SVM, the linear regression classifier (LRC) depends on the linear kernel matrix which is highlighted in Eq.(7) by designating 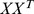 as 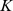 (same as in Eq.(5)). Differently from the SVM, the pseudo-inverse penalizes or removes the influence of singular vectors associated to singular values equal zero. If the matrix K is invertible, then+can be replaced by -1 in the last expression in Eq(7). We used the MATLAB command pinv for Eq.(7). Once the model is fitted the final decision function for this classifier is 
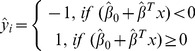
(8)where 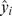 is the estimated label.

### Principal Component Space Analyses

Principal component analysis (PCA) is a powerful tool for dimension reduction that is often used in biomedical applications to deal with high-dimensional problems. After standardization of the data, we used the princomp MATLAB function to project the GM imaging data (matrix X) corresponding to CN and AD ADNI participants into principal component space. The performance of LRC, RLR and SVM are evaluated. To estimate the SVM we did not follow the kernel implementation described above, instead we used the LIBLINEAR library (version 1.8). It provides a fast implementation of the SVM with L_2_ regularization based on coordinate descent techniques [49]. Regularized methods in combination with PCA have been used before to predict stroke outcomes [50] and for imaging genetics analyses [51].

### Evaluation of classifier performance and parameter optimization

To evaluate classifier performance, in all cases we reserved a fixed dataset of 100 randomly selected participants (50 CN and 50 AD) for testing. Training datasets were randomly generated based on the remaining CN and AD participants. To estimate the optimal values of the regularization parameters both in voxel and PC spaces, we combined 10-fold cross-validations (CV) and grid search. For RLR we set the parameter 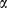 in Eq.(3) to 0.001. We then optimized the value of the parameter 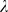 using the grid given by 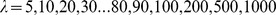 and for SV-SVM we used 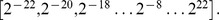 to tune the parameter C. For both methods at each grid point, the classifier was trained using 9 tenths of the training dataset and its performance was assessed using the fold left for testing by estimating the classification accuracy. We repeated this ten times using a different fold for testing and the rest of the data for training. We select the regularization parameters that produce maximum average accuracy across the 10 folds of the CV procedure. The classifier was then estimated using all the training dataset and the optimal value of the regularization parameter. Finally, the classifier's generalization capability was evaluated using the testing dataset. In the case of LRC, because there was no tuning of regularization parameters, we just fitted the model using the whole training dataset and its performance was evaluated using the testing dataset.

We computed overall classification accuracy to evaluate classifier performance as:
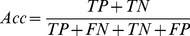
(9)where TP are AD patients correctly identified as AD, TN are controls correctly classified as controls, FN are AD patients incorrectly identified as controls and FP are controls incorrectly identified as AD.

### Evaluation of ill-posedness

In the context of discrete problems ill-posedness often translates into ill-conditioning of the linear kernel matrix. A common approach for evaluating the degree of ill-posedness for these problems is the study of linear kernel matrix conditioning via the use of the singular value decomposition(SVD) [32], [47]. The condition number is one measure often used to characterize matrix conditioning, which is computed as the ratio of the largest singular value to the smallest one. A matrix is singular if the condition number is infinite (singular values equal zeros are present) and ill-conditioned if its condition number is very large so its reciprocal approaches the machine's floating point precision [47]. We evaluated the influence of these factors on machine learning methods performance in the context of classification of structural MRI brain images.

### Evaluating the effect of dimension and sample size

The evaluation was based on the GM images of the CN and AD participants that were vectorized and used as samples to estimate the different classification models. For each sample size and dimension, computations were repeated 100 times for different samples of CN and AD participants selected at random. The imaging data were always normalized by subtracting the mean and dividing by the standard deviation in a voxel-wise fashion.

#### Experiment #1

We studied the impact of dimension and training dataset sample size in the voxel space on RLR, LRC and SM-SVM in terms of prediction performance (overall classification accuracy) when discriminating between GM images of AD and CN ADNI participants. The sample size was varied from 20 to 210 (20, 30, 40, 50, 60, 90, 120, 150, 180, 210) with balanced numbers of CN and AD participants. We varied the dimensions, given by the number of voxels included in the analysis, from 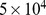 to 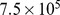 by selecting four different thresholds (0.86, 0.65, 0.2, and 0.0021) of the study-customized GM template.

#### Experiment #2

To study the conditioning of the linear kernel associated with classification of sMRI images using our ADNI dataset we estimated the singular values, rank and the condition numbers of all linear kernel matrices (K) across all samples sizes, dimensions and iterations tested in Experiment # 1.

#### Experiment # 3

We compared across 100 iterations the prediction performance of RLR, LRC, and SVM both in voxel and PC spaces for a very large dimension (D = 750 K voxels) and a large but fixed sample size (210). To select the optimal number of components for each iteration, we estimated classification accuracy for different sets of components generated by adding one component at a time, starting from the first component up to the total number of components. The maximum value of accuracy obtained across different sets of components was taken as the final result for a given iteration.

## Results

**Figure 1:**
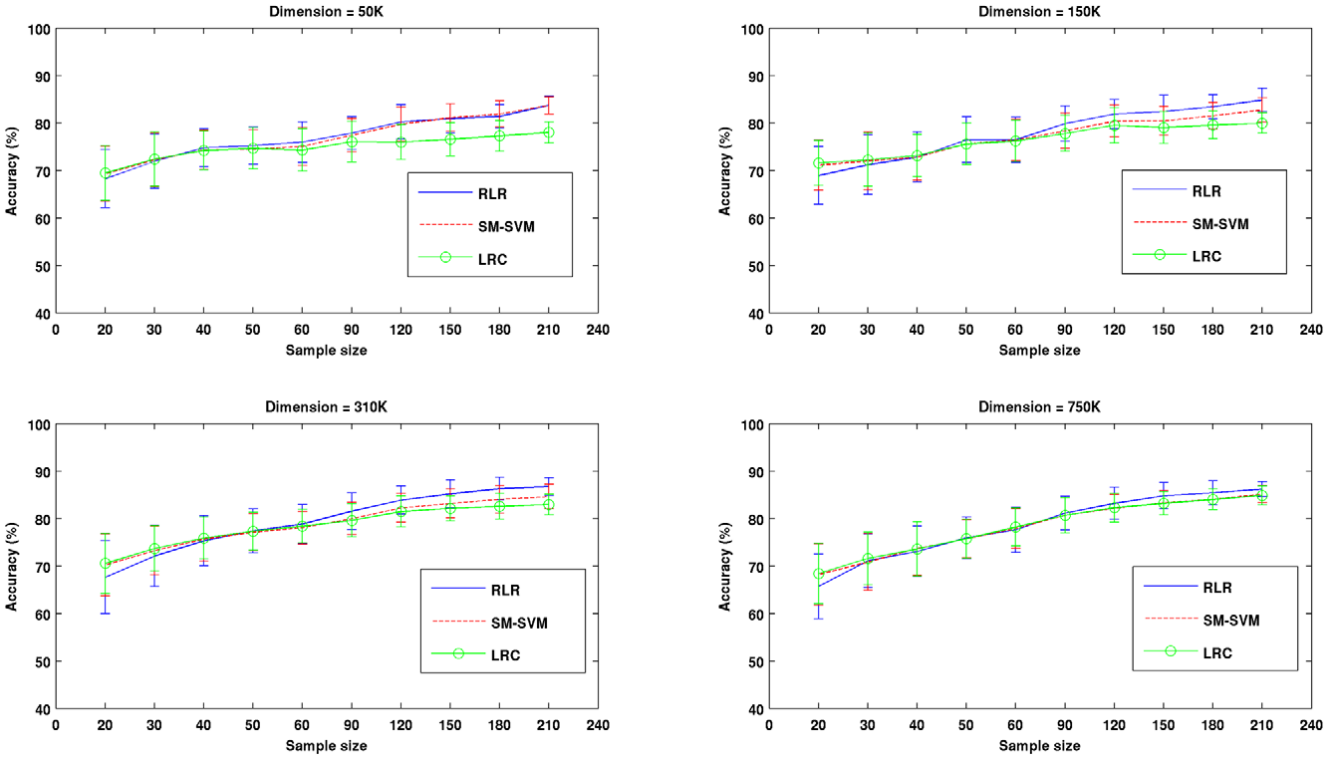
Results of Experiment # 1. The dependence of classification accuracy on sample size is depicted for all sample sizes, dimensions and methods we tested. Each panel represents the results of the three methods for a different dimension (50 K, 150 K, 310 K and 750 K). Each curve represents the average performance of a specific method across 100 iterations; bars depict one standard deviation.

**Figure 2:**
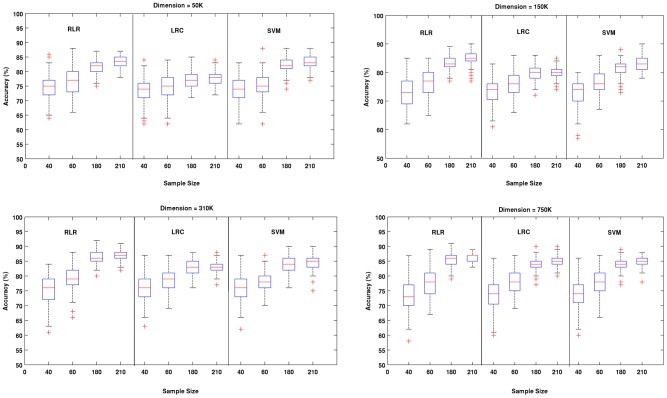
Results of Experiment # 1. More detailed information is provided for four sample sizes (40, 60, 180 and 210) using box plots. Each panel shows the behavior of the three methods across the selected sample sizes for a fixed dimension. For all three methods classification accuracy increased as the sample size increased. The performance of the two regularized methods (RLR and SVM) was in general very similar across all situations. Surprisingly, LRC was often very competitive, although it clearly performed worse for larger samples and lower dimensions.

**Figure 3:**
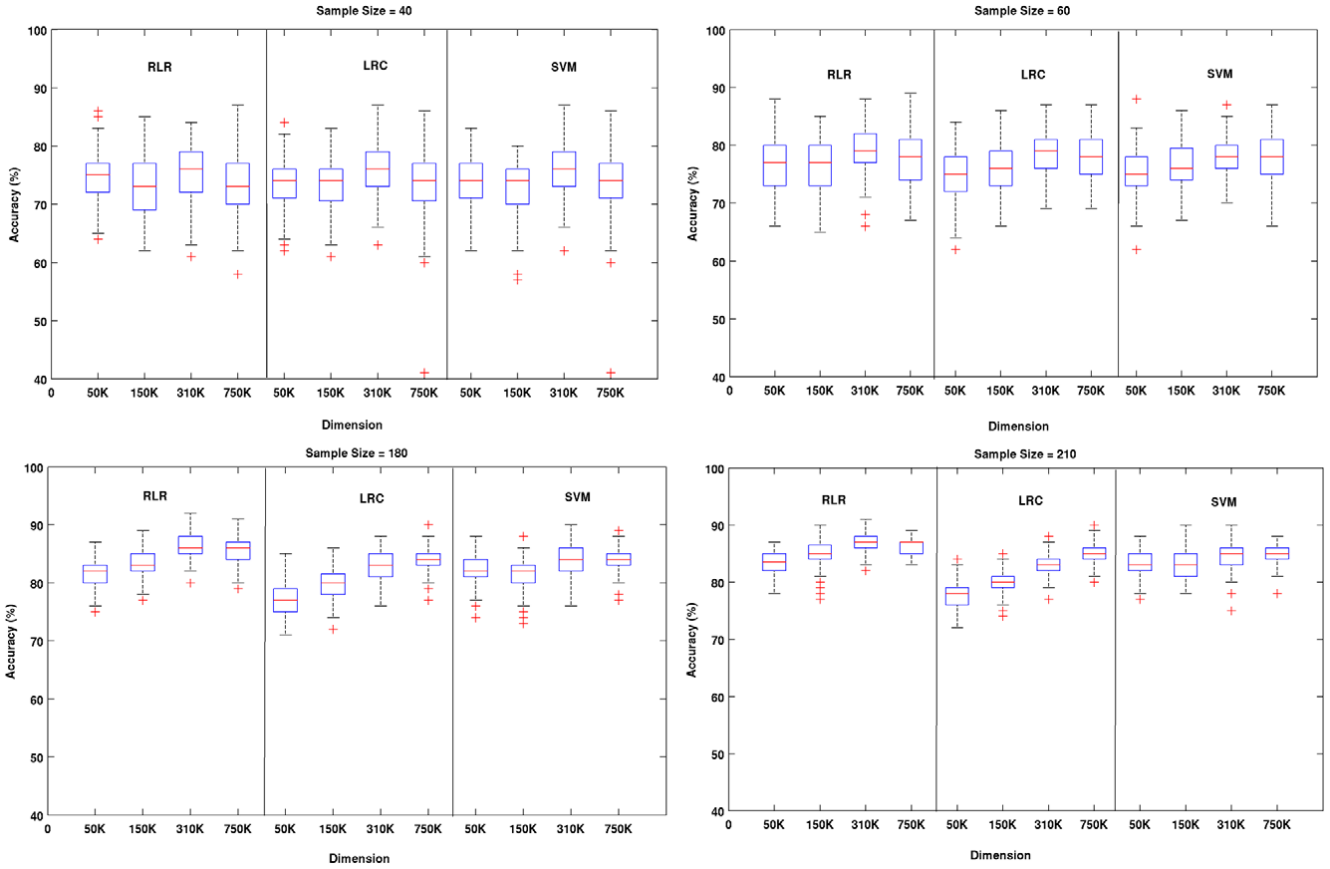
Results of Experiment # 1. An alternative view of the results in Figure 2 is presented by depicting in each panel the performance of the three methods across dimensions for a fixed sample size. It is clear that not only all methods were relatively robust to the increase of dimension, but also that their performance often improved. This was especially the case for the non-regularized LRC.

The results of the first experiment describing the impact of dimension and sample size in the voxel space on the RLR and SM-SVM performances are presented in Figures 1, 2 and 3. In Figure 1 the dependence of classification accuracy on sample size is depicted for all dimensions and methods we tested. Each curve represents the average performance across 100 iterations, with bars representing one standard deviation. In Figure 2 more detailed information is provided for four specific sample sizes (40, 60, 180 and 210) using box plots. Each panel shows the behavior of the three methods across the selected sample sizes for a fixed dimension. For the three methods classification accuracy increased as the sample size increased. Note this effect was attenuated for LRC in the smaller dimension. The performance of the two regularized methods (RLR and SVM) was in general very similar across all situations. Surprisingly, LRC was often very competitive, although it clearly performed worse for larger samples and lower dimensions. In Figure 3, the performance of the three methods across dimensions for a fixed sample size is shown in each panel. It is clear that all methods were relatively robust to the increase of dimension, and that very often they showed slight improvements in performance for larger dimensions especially for larger sample sizes. This last effect across dimensions was greater for the non-regularized LRC, especially for larger sample sizes.

**Figure 4:**
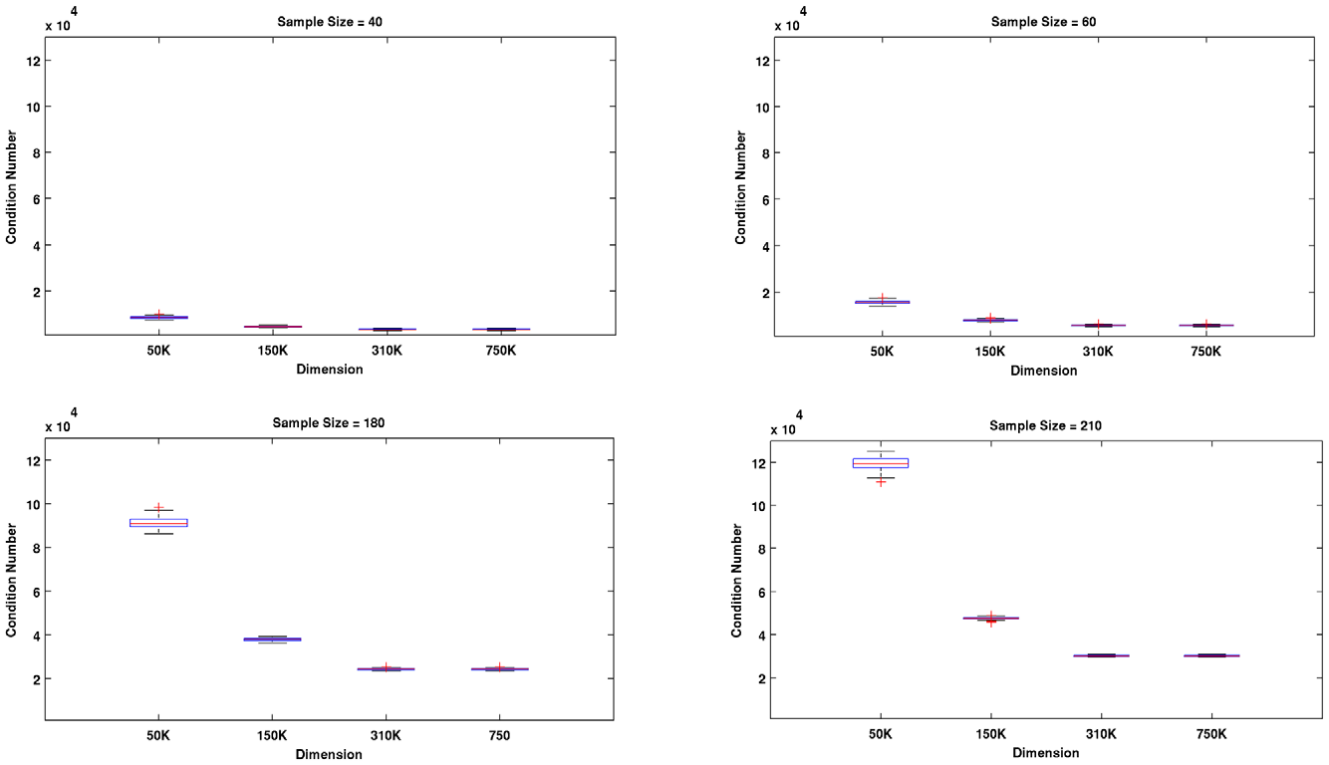
Results of Experiment # 2. For fixed sample size, improvements of the linear kernels matrices conditioning were observed as the dimension increased. The effect is more apparent for large sample sizes, when the difference in kernel's conditioning across dimensions was greatest. The worse conditioned kernel matrices were observed for larger sample sizes and the smallest dimension (50 K).

**Figure 5:**
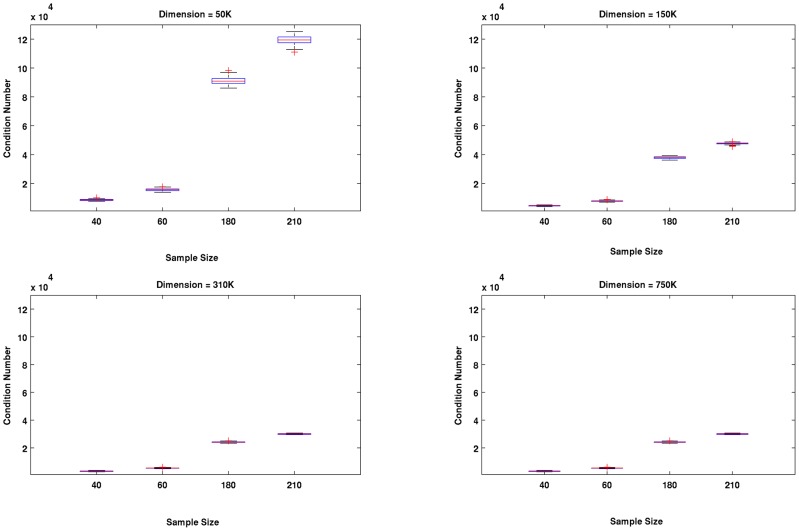
Results of Experiment # 2. This figure highlights that for a fixed dimension increases in sample sizes led to poorer conditioning of the kernels matrices (especially when the dimension was small) while at the same time as we observed in previous figures, it led to increases in classification accuracy.

**Figure 6:**
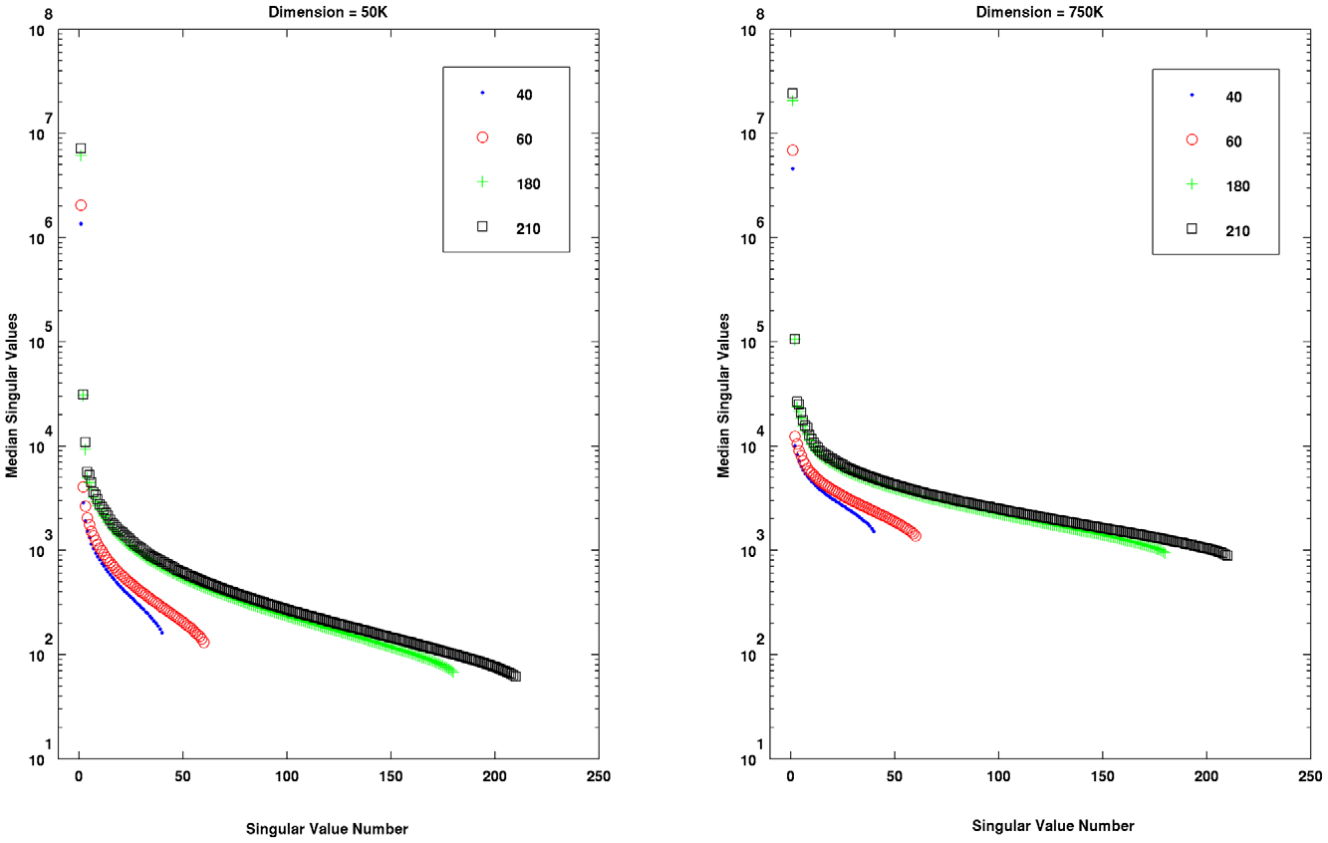
Results of Experiment # 2. The structure of singular values across selected sample sizes (40, 60, 180 and 210) and iterations is shown for two dimensions (50 K and 750 K). The median values of the singular values for the 100 iterations are plotted in logarithmic scale. The additional information brought by the increase of sample size was reflected by patterns of greater singular values when sample sizes were large, with the exception of the singular values located towards the “tails”, which caused poorer kernel matrices conditioning especially for the smaller dimension (50 K).

Most of the results of the second experiment are shown in Figures 4, 5 and 6. We found that in all cases (dimension-sample combinations) and iterations, the linear kernels matrices were full rank and well-conditioned. The observed patterns of conditioning shed light on the behavior of the linear classifiers tested in the first experiment. For example, a careful examination of the corresponding panels in Figures 3 and 4 reveals that for a fixed sample size, improvements in classifier performance with the increase of dimension was related to improvements of conditioning of the linear kernel matrices. The effect was more clearly observed for the larger sample sizes, for which the differences of kernel conditioning across dimensions were larger. The poorest conditioned kernel matrices occurred for larger sample sizes and the smallest dimension (50 K). This explains why the LRC clearly underperformed compared the other two methods in this situation (see Figure 1 upper left panel) but at the same time benefited the most with the improvement of conditioning of kernel matrices across dimensions. The regularization mechanism behind RLR and SVM reduced the detrimental effect of the singular vectors associated to the smaller singular values while the Moore-Penrose pseudo-inverse did not. It is designed to remove only the influence of singular values equal zero. One possible reason for the improvements of conditioning with the increase of dimension is the inclusion up to some point of a greater number of informative voxels which seems to be attenuated for the two larger dimensions we studied.

Figure 5 highlights that for a fixed dimension increases in sample sizes leads to worse conditioning (increase of condition numbers values) of the kernel matrices (especially for the smaller dimension). Simultaneously, it leads to increases in classification accuracy as seen in previous figures. A potential explanation is two opposing factors are associated with sample size increases. While increasing sample size provides additional information for discrimination between the two classes, it also worsens conditioning of the kernel matrices possibly via the introduction of collinearity across the rows of the kernel matrix. Regularized methods, as explained above, are capable of dealing with detrimental effects of poor conditioning of the kernel matrices. At the same time they take advantage of the additional information provided by the larger sample size, which explains their relatively greater increases in performance with respect to LRC when the sample size increased in the lower dimensional case (50 K).

In general, the ill-conditioning effects we discuss are very mild since all the kernel matrices were full rank and the reciprocal of their condition numbers were very far from the machine's precision. However, effects due to differences in conditioning did exist, and they influenced the performance of these methods as we have shown. In Figure 6 we show the structure of singular values across selected sample sizes (40, 60, 180 and 210) for two dimensions (50 K and 750 K). The median of the singular values for the 100 iterations are plotted in logarithmic scale. The additional information provided by larger sample sizes generally produced larger singular values, except for those located towards the “tails” (far right side). These few smaller singular values result in poorer conditioned kernel matrices especially when dimensions are small (e.g. 50 K). Regularized techniques, which are relatively robust to the effects of these small singular values, gain greater advantages from increases of sample size.

**Figure 7:**
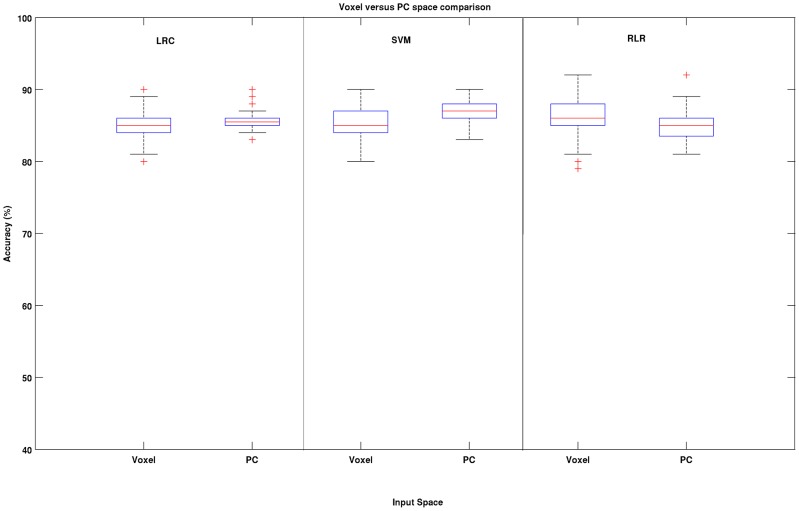
Results of Experiment # 3. The voxel space performances of the three methods are compared with their PC counterparts. We can see that, in general, relatively little or no gains were achieved by the three methods in the PC space. While LRC and the SVM showed slight improvements in performance, RLR showed slight decreases in performance.

The main results of the Experiment # 3 are presented in Figure 7. The voxel space performances of the three methods are compared with their PC counterparts. Despite the huge difference in the number of dimensions, relatively little or no gains were achieved by the three methods in the PC space. While LRC and the SVM showed slight improvements in performance in PC space the RLR showed slight decrease of performance.

## Discussion

This study provides a better understanding of the impact of dimension on linear classification methods that directly operate on high-dimensional neuroimaging spaces defined by voxels. It provides further evidence to dispel the common belief that due to the Curse of Dimensionality (CoD), feature selection is always necessary to achieve good performances in high-dimensional problem. Our research produced strong evidence about the robustness of several linear classifiers to increased dimensionality (up to 750 K voxels) in the context of sMRI data classification.

Some researchers have pointed out that the effect of the CoD on a machine learning algorithm depends on how it deals with sample neighborhoods [43]. Classifiers such as k-nearest or local kernel methods that operate on local neighborhoods to produce function estimates are more vulnerable to CoD effects, since in high-dimensional spaces the neighborhoods are no longer local. Cherkassky and Mulier noted that dimensionality alone is not necessarily a good measure of function complexity [23]. They suggested that it is necessary when characterizing function complexity to take into account both smoothness and dimensionality. Our work provides evidence that the impact of dimension on performance of machine learning methods depends on the problem's degree of ill-posedness. We observed, in classifying sMRI images, that linear classifiers were robust to the increase of dimension. This behavior is very likely related to the fact that unlike other types of data, structural MRI from CN and AD subjects are characterized by a large number of informative and correlated voxels, which leads in practice to well-conditioned linear kernel matrices. The problem we are studying here is strongly underdetermined, breaking the first (uniqueness) of the three Hadamard properties of well-posed problems and therefore is an ill-posed problem. On the other hand, it is characterized by relatively well-conditioned kernel matrices leading to good stability (third Hadamard's property). Lack of stability is perhaps the more complicated feature of an ill-posed problem. It implies that small changes in data (e.g. due to noise, etc) can lead to very different solutions and therefore regularization is needed. The lack of stability in discrete linear problems is often associated to the structure of the linear kernel matrices singular values and the presence of very small singular values [31], [32]. Regularization techniques are designed to dampen or filter out the effects of small singular values. The classification problem we studied here is stable although mild effects on the linear classifiers performance due to differences in conditioning of kernel matrices related to different sample sizes and dimensions are still observed. As should be expected the non-regularized LRC is the most affected by these effects. Interestingly, Hastie and colleagues have noted that another non-regularized technique the HM-SVM often shows similar performance to its regularized counterpart SM-SVM in high dimensional problems [43](page 658). HM-SVM works relatively well when classifying AD MRI images in the voxel space as reported before in the neuroimaging literature [14], [18], [52]. In additional experiments we noted similar patterns of performance of HM-SVM and LRC when compared to regularized methods (See upper left panel in Figure S1). In situations of worse kernel conditioning (lower dimension and larger sample sizes) they both underperformed regularized techniques. This suggests that the good performance of HM-SVM in high-dimensional problems is related to the well-conditioning of the associated linear kernel matrices.

It is striking how competitive the LRC performed (especially for the higher dimension studied here) with the much more sophisticated regularized methods, such as RLR and SVM. These results are possibly related to a phenomenon characteristic of high dimensional spaces called “data piling”, recently discovered and studied [53], [54]. For many linear classifiers, it manifests when most samples of one class project onto the same point of the separating hyperplane direction vector and when different classifiers yield the same direction vector. If two linear classifiers yield the same direction vector of the separating hyperplane they will produce the similar performance. Anh, Marron and their colleagues have proposed the use of the maximal data piling direction to improve performance of linear classifiers in high dimensional spaces [54], [55]. Our empirical work suggests that the data piling effect could be associated with less need of regularization due to the well conditioning of the linear kernel matrices. The three classifiers' performances were most similar when linear kernel matrices had best conditioning (e.g. larger dimensions). Interestingly, in a previous functional MRI study [10], similar performance of LRC, L_2_ regularized logistic regression and linear SVM was reported, with much smaller dimensions, fixed sample size and no optimization of regularization parameters.

There are several limitations in our study. We have evaluated the impact of dimension in a specific manner: by changing the threshold of the DARTEL study customized GM template, we generated problems of different number of variables. Although other ways of evaluating the effect of dimension could be devised, the approach chosen here naturally appears in the problem. In a GM voxel space analysis, we would first introduce the voxels with higher probability of being located in the GM tissue since it has been reported by several groups [17], [33], [41] that GM is more informative for brain MRI classification and regression than WM and CSF. By decreasing the threshold in GM images, we will at some point very likely including less informative or noisy voxels. Although we used elastic net regularization, it was not done with an optimal choice of both regularization parameters for computational reasons. We fixed in advance one of the regularization parameters (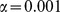) and optimized the second. We have observed in practice that this choice works well avoiding the heavy computational burden related to the optimization of both parameters. Finally, we focused our work on discrimination between CN subjects and AD patients and we did not attempt classification analyses using MCI data. The focus of our research is to develop biomarkers based on imaging data. Several biomarkers proposed before using machine learning methods such as SPARE-AD and STAND [3], [40] are metrics related to classifiers trained with well characterized databases of CN and AD subjects. Thus, in pursuing this goal it is important to develop classification methods highly discriminative of CN individuals from AD patients using sMRI and other types of information. We have recently proposed the use of the class-conditional probabilities associated to RLR study in work as a new biomarker for early prediction of AD [17], [56]. Importantly, a problem for this approach is that the ADNI cohort was not designed as a diagnostic clinical classification study to provide a realistic clinical testing ground. Thus, the enrolled cohort represents typical cases rather than the difficult diagnostic problems that clinicians have to face in practice [57].

Finally, we are not arguing that feature selection is not necessary or useful. Nor have we claimed that the methods studied here are the best solution to the problem of sMRI brain images classification. Our point is that, in some problems as the one we studied the commonly assumed devastating effect of dimension on classifiers performance is not necessarily present. The huge differences in dimensions between the voxel and PC spaces were not reflected in large differences in performance of these three methods. There are different factors that can influence the impact of dimension on a machine learning algorithm as for example the ill-posedness of the problem defined by the nature of the data. Therefore, the usefulness of feature selection in general will depend on the nature of the specific problem, employed machine learning algorithm, signal to noise ratio (e.g., sample size, etc) of the data, and other factors.

## Supporting Information

Figure S1 The performances of a hard margin (HM)-SVM and SM-SVM were compared across sample sizes and two dimensions 50 K and 750 K using a similar setup as in Experiment # 1. The HM-SVM shows, in general, a similar behavior to its regularized counterpart, although in situations of worse conditioning of the kernel matrices in this study (50 K and larger samples sizes) it underperforms in a similar fashion as the LRC does. The HM-SVM was implemented by setting the parameter C to 10^6^.
(TIF)
