# Supplementary figures and images for: Classification of Structural MRI Images in Alzheimer's Disease from the Perspective of Ill-Posed Problems
Source: PLoS One. 2012 Oct 10;7(10):e44877. doi: 10.1371/journal.pone.0044877 (PMC3468621; doi:10.1371/journal.pone.0044877)

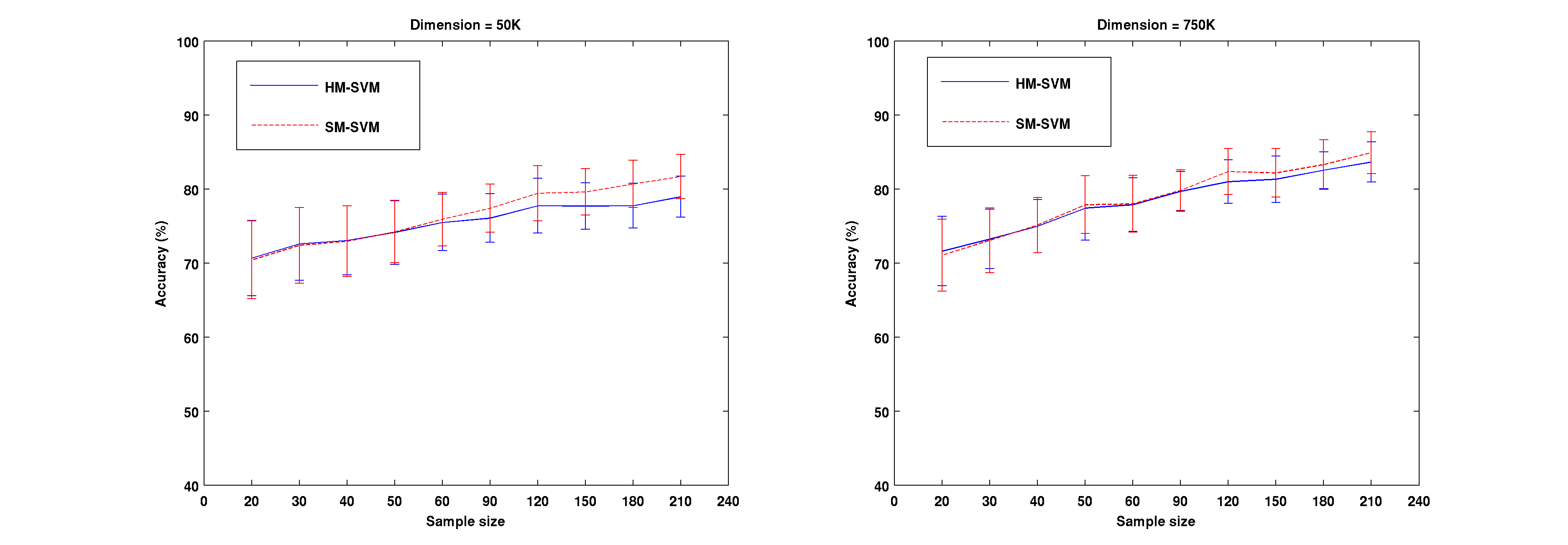

Supplement: Figure S1 — The performances of a hard margin (HM)-SVM and SM-SVM were compared across sample sizes and two dimensions 50 K and 750 K using a similar setup as in Experiment # 1. The HM-SVM shows, in general, a similar behavior to its regularized counterpart, although in situations of worse conditioning of the kernel matrices in this study (50 K and larger samples sizes) it underperforms in a similar fashion as the LRC does. The HM-SVM was implemented by setting the parameter C to 106. (TIF) [file pone.0044877.s001.tif]
